# Patient activation levels in cardiovascular disease: a cross-sectional study in Brazilian community pharmacies

**DOI:** 10.1590/1516-3180.2024.0411.R1.13062025

**Published:** 2025-10-06

**Authors:** Fabianna Marangoni Iglecias, Eduardo Riano, Francisco Javier Ferreira-Alfaya, Maria Isabel Valverde-Merino, Manuel Gomez-Guzman, Celia Piquer-Martinez, Maria José Zarzuelo

**Affiliations:** I Pharmacist, Ph.D. student, Department of Pharmacy and Pharmaceutical Technology, Faculty of Pharmacy, University of Granada, Granada, Spain.; II Pharmacist, Ph.D. student, Department of Pharmacy and Pharmaceutical Technology, Faculty of Pharmacy, University of Granada, Granada, Spain.; III Pharmacist and Professor, Department of Pharmacy and Pharmaceutical Technology, Faculty of Pharmacy, University of Granada, Granada, Spain.; IV Hospital Pharmacist Professor, Department of Pharmacy and Pharmaceutical Technology, Faculty of Pharmacy, University of Granada, Granada, Spain.; V Associate Professor, Department of Pharmacology, Faculty of Pharmacy, University of Granada, Granada, Spain.; VI Pharmacist. Department of Pharmacy and Pharmaceutical Technology, Faculty of Pharmacy, University of Granada, Granada, Spain.; VII Associate Professor, Department of Pharmacy and Pharmaceutical Technology, Faculty of Pharmacy, University of Granada, Granada, Spain.

**Keywords:** Self care, Pharmacists, Cardiovascular diseases, Brazil, Patient activation, Patient activation measure, Chronic disease management, Community pharmacy, Heart disease patient education

## Abstract

**BACKGROUND:**

Preventable cardiovascular diseases are among the leading causes of death in individuals aged < 70 years in Brazil.

**OBJECTIVE:**

This study assessed the level of patient activation among individuals with cardiovascular disease in Brazilian community pharmacies.

**DESIGN AND SETTING:**

This cross-sectional study included 348 Brazilian participants diagnosed with hypercholesterolemia and/or hypertension.

**METHODS:**

The Patient Activation Measure (PAM-13) questionnaire was used. In addition, sociodemographic and clinical variables were collected, including coronary risk evaluation and quality of life assessment. Student’s **t**-test was used to compare baseline quantitative variables between groups, and the chi-square test was used to assess associations for categorical variables. Pearson’s correlation was used to examine the relationships among the quality of life, clinical variables, sociodemographic data, and activation levels.

**RESULT:**

Participants had an average age of 59.0 ± 16.7 years and a low to moderate risk. The mean patient activation level was 2.8 out of 4, with high self-care responsibility and treatment adherence but lower confidence in maintaining lifestyle changes. Factors linked to lower activity included low physical activity (P < 0.001), multiple chronic conditions (P = 0.003), smoking (P = 0.016), age > 65 years (P = 0.033), low quality of life (P < 0.001), and high CVR (P < 0.001).

**CONCLUSION:**

Patient activation in cardiovascular care in the Brazilian population is positively affected by lifestyle factors, particularly physical activity. Intervention strategies that promote lifestyle changes can enhance patient activity and improve health outcomes in this population.

## INTRODUCTION

 The data from the World Health Organization (WHO)^1^ stated that non-communicable chronic diseases (NCDs) were responsible for the deaths of 17.7 million individuals worldwide in 2023, and accounted for approximately 30.8% of premature deaths in Brazil in 2019.^2^

 These diseases encompass a broad spectrum of chronic conditions typically linked to multiple causal factors. They are characterized by gradual onset, uncertain prognosis, and prolonged or indefinite duration, with a fluctuating clinical course involving durations of exacerbation and potential disabilities.^3^ The World Health Organization (WHO) stated that NCDs, including cardiovascular diseases (CVDs), are typically chronic and arise from a combination of genetic, physiological, environmental, and behavioral factors. Modifiable risk factors such as tobacco use, physical inactivity, alcohol consumption, and unhealthy diet significantly increase the likelihood of mortality from these diseases.^4^

 Healthcare systems face mounting challenges as the prevalence of chronic diseases continues to increase, both from the economic burden and limited capacity to manage these conditions effectively, particularly in the short term.^5^ Evidence suggests that actively engaging patients in their healthcare can help maintain a balance between individual health outcomes and the sustainability of the healthcare system.^6^

 Patient activation, as defined by Hibbard et al.,^7^ refers to an understanding of their role in the care process and their knowledge, skills, and confidence in managing their health and healthcare. Active patients have the skills and behavioral repertoire to manage their illness, maintain their health functioning, prevent health deterioration, collaborate with their healthcare providers, and access appropriate, high-quality care. 

 The central role of patients in decision-making and in managing their care is increasingly being recognized.^8^ This concept of activated patients is fundamental to the patient care experience. Increased patient activation is associated with improved health outcomes in several long-term chronic conditions, including reduced premature mortality and hospitalization.^7^

 Understanding a patient’s level of activation is essential for determining the best strategy to empower them in self-management of their disease, including reducing cardiovascular risk (CVR) through behavioral changes.^9^ The majority of patients fall within Stage 3, where they have the necessary medical knowledge to take action; however, they sometimes lack the skills or confidence to adopt new health behaviors.^10,11^ Public policies can be aligned with activation levels, directing resources toward vulnerable populations, such as those with low educational attainment, and supporting patients in developing the knowledge necessary for responsible health autonomy.^9^

 One approach to bridge the gap in effective NCD management and reduce the burden on the healthcare system is to involve community pharmacists as key facilitators in promoting patient activation and exploring this condition. 

## OBJECTIVE

 This study explored the activation levels in patients with CVD in Brazil. 

## METHODS

### Study design

 A cross-sectional study designed to assess patient activation levels using the PAM-13 questionnaire,^7^ was conducted using an adapted and validated version for the Brazilian population.^12^ The questionnaire and variables were administered and recorded by the research pharmacist. 

### Study population

 Patients were recruited from urban community pharmacies (n = 13) throughout the country, except Amazonia, and were invited to participate voluntarily. Invitations to community pharmacists were distributed via an email campaign supported by the Pharmacy Association (Anfarmag), and pharmacists were directly recruited. 

 Participation was contingent on meeting the specific inclusion and exclusion criteria. Eligible participants were adults aged 18 years or older who were receiving treatment for hypertension and/or hypercholesterolemia and provided signed informed consent. Patients who did not provide informed consent or those who had a disability that prevented them from completing the PAM-13 questionnaire^7^ were excluded. 

**Figure 1:**
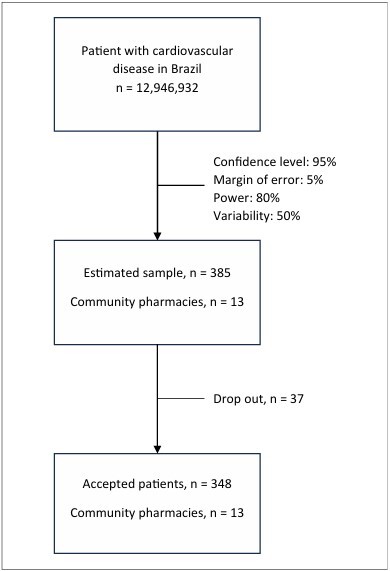
Study flowchart.

 The sample size was calculated using the following formula: $n=\frac{N*Z^{2}*p*\left(1-p\right)}{\left(N-1\right)+e^{2}+Z^{2}*\left(1-p\right)}$ ,where *n* is the sample size, N is the total population, Z is the confidence level value (1-α) at 95%, P value is the expected proportion in the population, and e is the absolute precision. With a 5% margin of error, 80% power, and a known variability of 50%, the populations of patients with CVD in Brazil were considered according to Cardiovascular Statistics–Brazil 2020 (12,946,932).^13^ The estimated sample size was 385 patients, accounting for a potential 20% dropoutrate (Figure 1). 

### Variables

**Patient activation:** Patient activation was measured using the PAM-13 questionnaire^7^ (Insignia Health 1667562221). This 13-item tool assesses the belief that the patient’s role is crucial, the confidence and knowledge necessary to take action, the implementation of actions to maintain and improve one’s health, and the ability to stay committed to these actions even under stress.^12,14^ Individual item scores range from 0 to 4 (0 = not applicable, 1 = strongly disagree, 2 = disagree, 3 = agree, and 4 = strongly agree) on a Likert Scale. The average PAM-13 score is converted into a total score ranging from 0 to 100, with higher scores indicating greater activity. Based on the Insignia Health guidelines,^15^ PAM-13 scores were categorized into one of four activation levels. Level 1 corresponded to very low activation (0–47.0 points), indicating disengagement and feeling overwhelmed, Level 2 (47.1–55.1) represented becoming aware but still struggling, Level 3 (55.2–67.0) indicated taking action, and Level 4 (67.1–100) represented high activation, implying sustaining behaviors and continuing to improve.^7^

**Quality of life:** Quality of life refers to the evaluation of an individual’s overall well-being and life satisfaction as influenced by or related to their health status. This was measured during the patient visit using a visual analog scale (VAS). The values ranged from 0 to 100, with 0 indicating the worst state of health and 100 indicating the best. 

**Sociodemographic variables:** Age, sex, educational level, autonomy status, employment status, and health-related variables (comorbidities, smoking status, physical activity, and disease duration) were self-reported. 

**Clinical variables:** Clinical variables were measured by the research pharmacist using blood pressure values measured by a sphygmomanometer in mmHg and total cholesterol values by an analysis performed by a doctor or in pharmacies with the equipment for measurement in mg/dL. 

**CVR factors:** The CVR factors were measured using Systematic Coronary Risk Evaluation (SCORE). This table assesses CVR by calculating the probability of developing death of cardiovascular origin within 10 years according to sex, smoking status, age, systolic blood pressure, and total cholesterol. Increased CVR is considered to be higher than ≥ 5%. 

### Statistical analysis

 Data were analyzed using SPSS version 28.0 (SPSS Inc., Chicago, Illinois, United States). Descriptive statistics, including frequency tables for qualitative variables and measures of central tendency (mean and standard deviation) for quantitative variables, were generated to characterize the study sample. 

 Student’s *t*-test for independent samples was used to compare baseline quantitative variables between the different groups. For categorical variables, the chi-square test was used to assess the associations. Multivariate generalized ordinal logistic regression was used to compare the associations of sociodemographic factors and CVR with different PAM-13 levels. Pearson’s correlation coefficients (r) were calculated to examine the bivariate relationships among quality of life, clinical variables, sociodemographic data, and activation levels. 

### Ethic approval

 This study was conducted following the resolutions in force in Brazil on Research Ethics (Resolution CNS 466/12), although the indications and treatment of subjects participating in the study followed the same practical clinical routine, and no interference was present with them during the study. Similarly, the study was developed according to a protocol and based on procedures that guarantee compliance with ICH/BPC (International Harmonization Conference/Good Clinical Practice) standards. This project was approved by the Brazil Research Ethics Committee (CAAE: 52996521.9.0000.5553) on December 27, 2022. 

## RESULTS

 The study included 348 patients with a mean age of 59.0 ± 16.7 years. An analysis of CVR revealed that the average blood pressure was 129.6 ± 19.1 mmHg for systolic pressure and 69.7 ± 17.2 mmHg for diastolic pressure. The mean total cholesterol level was 205.4 ± 38.0 mg/dL. The CVR assessed using the SCORE scale yielded a mean value of 2.8 ± 2.7, classified as having low to moderate CVR. The mean number of medications taken by patients was 4.5 ± 3.3, and the overall quality of life for the study population was assessed at 71.6 ± 19.7 out of 100 points (Table 1). 

**Table 1:** Sociodemographic and cardiovascular data

**Variable**	**Mean ± SD (min-max)**
Age (years old)	59.0 ± 16.7
Systolic blood pressure (mmHg)	129.6 ± 19.1 (90-211)
Diastolic blood pressure (mmHg)	69.7 ± 17.2 (10-102)
Total cholesterol (mg/dL)	205.4 ± 38.0 (85-330)
SCORE* (low/moderate)	2.8 ± 2.7 (0-12)
Number of medications per patient	4.5 ± 3.3 (0-15)
Quality of life	71.6 ± 19.7

SCORE = Systematic Coronary Risk Evaluation;

* SCORE is analyzed as a continuous variable based on its numerical score (mean and standard deviation) and is categorized as low or moderate according to established thresholds.

 Most participants were women, comprising 61.1% (n = 212) of the sample. Regarding educational attainment, 31.9% (n = 111) of the patients had no formal education, whereas 17.8% (n = 62) had completed university-level education. In addition, 51.7% of participants (n = 180) were retired. Of the patients, 79.0 % (n = 275) were non-smokers, and 56.3% (n = 196) engaged in a certain form of physical activity, either occasionally or daily. A total of 63.2% (n = 220) had multiple comorbidities, with 57.2% (n = 199) having these conditions for more than 10 years. In terms of living arrangements, the vast majority of patients (79.0%, n = 275) were autonomous and lived with a companion, 12.6% (n = 44) lived alone independently, and 8.4% (n = 29) required caregivers (Table 2). 

**Table 2:** Descriptive statistics

**Variable**	**n (%)**	**Level 1 (≤ 47.0) (n = 61)**	**Level 2 (47.1-55.1) (n = 57)**	**Level 3 (55.2-67.0) (n = 123)**	**Level 4 (≥ 67.1) (n = 107)**	**P value**
Gender (female)	212 (61.1)	33 (45.9)	44 (77.2)	72 (59.0)	63 (58.9)	0.039*
Educational level (no education)	111 (31.9)	30 (39.3)	19 (50.0)	35 (28.9)	2 (23.8)	0.009*
Autonomy status (autonomous with companion)	275 (79.0)	55 (68.9)	23 (66.7)	99 (85.0)	98 (84.6)	0.628
Employment status (retired)	180 (51.7)	23 (26.3)	21 (34.6)	64 (38.4)	72 (44.3)	0.466
Smoking status (non-smoker)	275 (79.0)	47 (57.1)	29 (83.3)	102 (87.5)	97 (84.6)	0.022*
Physical activity (yes)	196 (56.3)	17 (21.4)	12 (33.3)	82 (70.0)	85 (74.4)	< 0.001*
Multiple conditions (yes)	220 (63.2)	63 (78.6)	32 (91.7)	78 (67.5)	47 (41.0)	< 0.001*
Duration of disease (over 10 years)	199 (57.2)	53 (64.3)	29 (83.3)	50 (42.5)	67 (59.0)	0.049*
SCORE** (low/moderate)	270 (77.6)	42 (44.4)	21 (66.7)	124 (88.9)	83 (100.0)	< 0.001*

SCORE = Systematic Coronary Risk Evaluation.

* P < 0.05. Chi-square test.

** SCORE is analyzed as a continuous variable based on its numerical score (mean and standard deviation) and categorized as low or moderate according to established thresholds.

 The patients had a good level of activation, with the majority between levels 3 (35.3%, n = 123) and 4 (30.7%, n = 107). Specifically, 17.5% of the patients were classified as Level 1, 16.4% as Level 2, 35.3% as Level 3, and 30.7% as Level 4 (Table 2). When categorized by PAM-13 level, a significant difference was observed in Level 2, with 77.2% of women (P = 0.039) with a low level of education; only 23.8% of patients were found in level 4 (P = 0.009). Moreover, 84.6% of patients who did not smoke and 74.4% of those who engaged in regular physical activity were at Level 4 (P = 0.022 and P < 0.001, respectively). In addition, presenting with multiple pathologies, 91.7% of the patients were at activation level 2 (P < 0.001), as were 83.3% of the patients who had been ill for more than 10 years (P = 0.049). Finally, 100.0% of patients with low/moderate CVR had an activation level of 4 (P < 0.001) (Table 2). 

 The results of the PAM-13 questionnaire^7^ indicated a total score of 63.9 ± 19.6 out of 100, with a mean activation level of 2.8 ± 1.1 out of 4. The best-scored questions were question 1 ("After all, I am responsible for taking care of my health") 3.5 ± 0.7 out of 4 and question 7 ("I am sure that I can continue the medical treatment correctly at home") 3.5 ± 0.7, with the highest percentage of responses in complete agreement, 60.6% and 64.3%, respectively. In contrast, the lowest-scoring questions were question 13 ("I am sure that I can maintain my lifestyle changes, such as eating properly and exercising, even in times of stress") 2.7 ± 0.9 and question 9 ("I know the different treatment options for my health problems") 2.8 ± 1.0, with 8.6% and 10.4% of responses in total disagreement, respectively (Table 3). 

**Table 3:** Patient Activation Measure (PAM-13) questionnaire scores

**Question**	**Mean ± SD**	**Totally Disagree (%)**	**Disagree (%)**	**Agree (%)**	**Totally Agree (%)**
Q1. After all, I am responsible for taking care of my health	3.5 ± 0.7	2.4	6.3	30.7	60.6
Q2. Taking an active role in self-care is the most beneficial aspect of my health	3.4 ± 0.8	0.8	16.3	24.8	58.1
Q3. I am confident that I can take steps that will help prevent or reduce symptoms or problems related to my health	3.3 ± 0.8	1.6	13.3	36.7	48.4
Q4. I know what each of the medicines I have been prescribed are for	3.3 ± 1.0	8.6	9.4	25.0	57.0
Q5. I am confident that I am able to differentiate whether it is necessary to go to the doctor or whether I can solve the health problem myself	3.2 ± 0.9	4.0	16.0	35.2	44.8
Q6. I am sure I can tell the doctor about my concerns, even if he/she does not ask me	3.1 ± 0.9	6.3	20.3	28.9	44.5
Q7. I am sure that I can continue my medical treatment correctly at home	3.5 ± 0.7	2.3	7.8	25.6	64.3
Q8. I understand my health problems and their causes	3.2 ± 0.8	3.9	13.3	37.5	45.3
Q9. I am aware of the different treatment options for my health problems	2.8 ± 1.0	10.4	29.6	27.2	32.8
Q10. I have been able to maintain my lifestyle changes, such as eating right and exercising	3.0 ± 0.9	3.9	26.0	37.8	32.3
Q11. I know how to prevent health-related problems	3.1 ± 0.8	2.4	23.0	43.3	32.3
Q12. I am confident that I can find solutions when new health-related problems arise	3.1 ± 0.9	4.2	20.8	40.8	34.2
Q13. I am confident that I can maintain my lifestyle changes, such as eating right and exercising, even in times of stress	2.7 ± 0.9	8.6	35.2	33.6	22.7
Total score	63.9 ± 19.6				

 Statistically significant correlations were observed between variables such as age, smoking, educational level, having more than one pathology, cardiovascular risk (SCORE), medication count, physical activity, and quality of life, with the vast majority of the questions of the PAM questionnaire (Table 4). A negative correlation among age, smoking habit, multiple conditions, CVR, and medication count, and a positive correlation with physical activity and quality of life with the item that considered oneself responsible for taking care of one’s health (P < 0.05 and P < 0.001) were observed. Similarly, a negative correlation among age, educational level, multiple conditions, CVR and medication count and a positive correlation with physical activity and quality of life with developing an active role in self-care, which was observed as the most beneficial aspect of one’s health (P < 0.05 and P < 0.001) were observed. A negative correlation among age, educational level, multiple conditions, CVR, and medication count, and a positive correlation with physical activity and quality of life, with confidence in taking measures that would help prevent or reduce symptoms or health-related problems (P < 0.05 and P < 0.001) were observed. In addition, knowing the purpose of each prescribed medication correlated negatively with age, smoking habit, educational level, multiple conditions, CVR, and medication count, and positively correlated with physical activity and the quality of life (P < 0.05, P < 0.001). Age, educational level, multiple conditions, CVR, and medication count and positively correlated with physical activity and quality of life, with the ability to differentiate when it is necessary to visit a doctor versus solving a health problem independently (P < 0.05 and P < 0.001). Less smoking led to fewer medications prescribed and a higher quality of life (P < 0.05), and the more confident the patients were that they could inform their doctors what was bothering them, even if they were not asked. Furthermore, confidence in correctly following medical treatment at home was higher in younger patients (P < 0.001), those with a higher educational level (P < 0.05), fewer comorbidities (P < 0.001), lower CVR (P < 0.001), fewer medications prescribed (P < 0.001), and higher physical activity and quality of life (P < 0.001). Understanding their health problems and causes was significantly correlated with SCORE (P < 0.05), medication count, physical activity, and quality of life (P < 0.001). Knowledge of different treatment options for health problems was higher in younger patients (P < 0.05), non-smoking patients (P < 0.001), and those with lower CVR (P < 0.05), less prescribed medication (P < 0.001), more physical activity (P < 0.001), and higher quality of life (P < 0.05). Maintaining lifestyle changes, such as eating well and exercising, was negatively correlated with smoking, education level, concomitant diseases, CVR, and number of prescribed medications (P < 0.05), and positively correlated with physical activity and quality of life (P < 0.001). Understanding how to prevent health-related issues significantly correlated with age, multiple conditions, SCORE, medication count, physical activity, and quality of life (P < 0.05, P < 0.001), and the ability to find solutions when new health problems arose exhibited negative correlations with age and SCORE (P < 0.05) and positive correlations with the quality of life (P < 0.001) (Table 4). 

**Table 4:** Significant correlations between variables and the Patient Activation Measure-13 questionnaire, quality of life, and activation level

**Question**	**Age (-)**	**Smoke (-)**	**Educational level (+)**	**Multiple Conditions (-)**	**SCORE (-)**	**Medication Count (-)**	**Physical Activity (+)**	**Quality of live (+)**
Q1. After all, I am responsible for taking care of my health	0.253*	0.290**		0.302**	0.485**	0.308**	0.274*	0.315**
Q2. Taking an active role in self-care is the most beneficial aspect of my health	0.214*		0.175*	0.268*	0.605**	0.420**	0.367**	0.366**
Q3. I am confident that I can take steps that will help prevent or reduce symptoms or problems related to my health	0.322**		0.247*	0.338**	0.580**	0.505**	0.497**	0.506**
Q4. I know what each of the medicines I have been prescribed are for	0.307**	0.234*	0.290**	0.250*	0.524**	0.361**	0.260*	0.274*
Q5. I am confident that I am able to differentiate whether it is necessary to go to the doctor or whether I can solve the health problem myself	0.366**		0.240*	0.194*	0.321*	0.228*	0.305**	0.246*
Q6. I am sure I can tell the doctor about my concerns, even if he/she does not ask me		0.229*				0.184*		0.195*
Q7. I am sure that I can continue my medical treatment correctly at home	0.301**		0.261*	0.294**	0.412**	0.428**	0.332**	0.375**
Q8. I understand my health problems and their causes					0.387*	0.281**	0.303**	0.300**
Q9. I am aware of the different treatment options for my health problems	0.209*	0.312**			0.380*	0.337**	0.293**	0.237*
Q10. I have been able to maintain my lifestyle changes, such as eating right and exercising		0.195*	0.190*	0.207*	0.354*	0.206*	0.361**	0.381**
Q11. I know how to prevent health-related problems	0.308**			0.226*	0.408**	0.306**	0.233*	0.345**
Q12. I am confident that I can find solutions when new health-related problems arise	0.184*				0.269*			0.422**
Q13. I am confident that I can maintain my lifestyle changes, such as eating right and exercising, even in times of stress		0.235*	0.200*	0.233*	0.309*	0.254*	0.375**	0.336**
Quality of life	0.209*		0.205*	0.189*	0.250*	0.380**		
Activation level	0.148*	0.251*	0.222*	0.313*	0.576**	0.434**	0.434**	

* P < 0.050;

** P < 0.001.

Correlations are classified as follows: (−) negative Pearson’s correlation (r < 0) and (+) positive Pearson’s correlation (r > 0).

SCORE = Systematic Coronary Risk Evaluation.

 We analyzed the correlations that could exist between the quality of life, level of activation, and level of CVR with different variables of the questionnaire and found that younger patients with university education, less concomitant pathology, fewer medications, and more physical activity had a better quality of life (P < 0.05, P < 0.001). In addition, younger patients who did not smoke, had fewer concomitant pathologies, lower CVR, fewer prescribed medications, and more physical activity had a higher level of activity (P < 0.05, P < 0.001) (Table 4). 

 The probability of having a low level of activation with low physical activity (OR = 3.87, 95%CI = 2.09–7.17, P < 0.001), having more than one pathology (OR = 1.48, 95%CI = 1.17–1.88, P = 0.003), smoking (OR = 1.64, 95%CI = 1.04–2.60, P = 0.016), being older than 65 years (OR = 1.25, 95%CI = 1.02–1.52, P = 0.033), having a low quality of life (OR = 0.96, 95%CI = 0.95–0.98, P < 0.001), and being at high CVR (OR = 3.47, 95%CI = 1.99–6.05, P < 0.001) was high (Table 5). In addition, performing occasional or daily physical activity was more likely to have a low/moderate CVR (OR = 2.69, 95%CI = 1.05–6.93, P = 0.039), controlled blood pressure (OR = 2.47, 95%CI = 1.15–5.31, P = 0.025), and fewer concomitant pathologies (OR = 1.42, 95%CI = 1.10–1.87, P = 0.012) (Table 5). 

**Table 5:** Logistic regression between patient activation level and physical activity and study variables

**Low level of activation**	**OR**	**95%CI**	**P value**
Low physical activity	3.87	2.09-7.17	< 0.001
Multiple condition	1.48	1.17-1.88	0.003
Smoke	1.64	1.04-2.60	0.016
Over 65 years	1.25	1.02-1.52	0.033
Low quality of life	0.96	0.95-0.98	< 0.001
High SCORE	3.47	1.99-6.05	< 0.001
**Physical activity**			
Low/moderate CVR	2.69	1.05-6.93	0.039
Controlled BP	2.47	1.15-5-31	0.025
Fewer concomitant pathologies	1.42	1.10-1.87	0.012

OR = odds ratio; CI = confidence interval; SCORE = Systematic Coronary Risk Evaluation; CVR = cardiovascular risk; BP = blood pressure.

## DISCUSSION

 We found that factors such as low physical activity, smoking, advanced age, high CVR, and low QoL were significantly associated with reduced levels of patient activation. These findings underscore the need for targeted interventions to enhance the activation among patients with these risk factors, particularly through lifestyle modification and supportive care strategies. 

 Several validated methods are available to assess different facets of patient activation, including health locus of control,^16^ self-efficacy in self-management behaviors,^17^ and readiness to change health-related behaviors.^18^ For example, the health locus of control has been used to explore the extent to which individuals believe they have control over their health outcomes, whereas self-efficacy measures assess the confidence of patients in executing specific health-related behaviors. The PAM-13 questionnaire^7^ was selected as a tool to assess activation in terms of an individual’s knowledge, skills, beliefs, and confidence in managing their health.^10,19^ A preference for taking an active role in healthcare can significantly enhance a patient’s ability to engage in shared decision-making with healthcare professionals.^20^ This dynamic is particularly relevant for patients with chronic diseases, where control preferences, health promotion educational intervention, active participation in decision-making, and increased activation have been demonstrated to improve outcomes.^21-23^

 Consistent with the findings of Magnezi et al.,^24^ our study revealed a positive correlation between patient activity and quality of life. Specifically, among patients with visual impairment, PAM-13 revealed significant correlations with activation and the quality of life. Similarly, the average PAM-13 score was 53.4 ± 13.8 in patients with chronic renal disease, with the majority (73%) exhibiting low activation. Notably, patient activation decreased significantly with age and increased with higher educational levels.^25^

 In studies on cardiovascular patients, such as that by Goevaerts et al.,^26^ the baseline mean PAM-13 score was 59.2 ± 9.5, with 65% of patients at Level 3. We observed slightly higher PAM-13 scores in patients with CVD, with only 35.3% reaching Level 3, indicating a distinct distribution. Similar scores were reported by Hernar et al.,^27^ who studied individuals at risk of early health deterioration, revealing an average PAM-13 score of 69.8 ± 14.8. Higher activation scores were generally associated with healthier behaviors across broader populations, including increased physical activity, a finding that aligns with our results, that demonstrated performing occasional or daily physical activity, it was more likely to have a low/moderate CVR, controlled blood pressure and fewer concomitant pathologies, and more level of activation. 

 Zang et al.^28^ reported that in older adults at an increased risk of CVDs in rural areas, education, multimorbidity, and a family history of CVD positively and age negatively influenced activation. In line with these results, patients with low activation levels had multimorbidity, were older than 65 years, and had a high CVR. 

 General practitioners perceive patient activation as a crucial factor in CVD prevention and report that overcoming the barriers to lifestyle counselling identified by family physicians is a prerequisite for effective patient-centered consultation on CVR factors. In addition, interprofessional collaboration with pharmacists could alleviate the burden on GPs and thus reduce these barriers.^29^ Overall, lower levels of activation are often associated with advanced age, poor health-related quality of life, greater decisional conflict, and reduced medication adherence.^30^ Patient activation is crucial for improving the quality of life among individuals with chronic diseases, as it encompasses patients’ motivation, knowledge, and ability to manage both acute crises and chronic symptoms. Increasing evidence suggests that patients with higher activation are better equipped to engage in self-care,^31^ which ultimately leads to improved health outcomes.^32^

 This study had several strengths. This study was conducted across all regions of Brazil except Amazonia, allowing for a broad generalization of our results. In addition, validated instruments were used to measure the patient activity, quality of life, and CVR. 

### Limitations

 We could not assess the temporal effects or determine causality due to the cross-sectional design of the study. Longitudinal studies are required to better understand the long-term influences on patient activation. The reliance on self-reported questionnaires may introduce potential misclassifications owing to socially desirable responses. 

 Variables such as waist circumference and body mass index were not measured due to opposition from several patients. Data such as complete lipid profile and glycosylated hemoglobin were not included because only one pharmacy had a measuring device, and insufficient data were obtained to ensure consistency in the statistical analysis. 

### Future directions

 Patients with CVD feel more responsible for their healthcare and are more confident in being able to correctly follow medical treatment at home. However, they are less confident in being able to maintain lifestyle changes and are less aware of the different treatment options available for their health problems. Pharmacists play a decisive role in improving patients’ understanding of their conditions, being able to actively involve them in their treatment, and transforming therapeutic success into personal achievement. 

 These findings suggest specific areas of strength and vulnerability in patient engagement, which may inform future research on tailored support strategies for individuals with CVD. 

## CONCLUSIONS

 The level of patient activation in managing cardiovascular health is influenced by multiple factors, including physical activity, age, presence of comorbidities, and overall quality of life. Patients who engaged in regular physical activity demonstrated higher activation levels, better blood pressure control, and a lower CVR, highlighting the significance of promoting physical activity as a strategy for enhancing patient self-management and improving health outcomes. These findings underscore the need for targeted interventions to enhance activation among patients with these risk factors, particularly through lifestyle modification and supportive care strategies. Interventions emphasizing physical activity, self-efficacy, and individualized support are recommended to enhance patient empowerment and engagement in healthcare. 

## References

[1] Islam A, Sultana H, Nazmul Hassan Refat M, Farhana Z (2024). The global burden of overweight-obesity and its association with economic status, benefiting from STEPs survey of WHO member states: A meta-analysis. Prev Med Rep.

[2] Malta DC, Gomes CS, Veloso GA (2024). Noncommunicable disease burden in Brazil and its states from 1990 to 2021, with projections for 2030. Public Health.

[3] GBD 2019 Chronic Respiratory Diseases Collaborators (2023). Global burden of chronic respiratory diseases and risk factors, 1990-2019: an update from the Global Burden of Disease Study 2019. EClinicalMedicine.

[4] GBD 2021 Stroke Risk Factor Collaborators (2024). Global, regional, and national burden of stroke and its risk factors, 1990-2021: a systematic analysis for the Global Burden of Disease Study 2021. Lancet Neurol.

[5] Hajat C, Stein E (2018). The global burden of multiple chronic conditions: A narrative review. Prev Med Rep.

[6] Dineen-Griffin S, Garcia-Cardenas V, Williams K, Benrimoj SI (2019). Helping patients help themselves: a systematic review of self-management support strategies in primary health care practice. PloS One.

[7] Hibbard JH, Stockard J, Mahoney ER, Tusler M (2004). Development of the Patient Activation Measure (PAM): conceptualizing and measuring activation in patients and consumers. Health Serv Res.

[8] Hibbard JH, Greene J (2013). What the evidence shows about patient activation: better health outcomes and care experiences; fewer data on costs. Health Aff.

[9] Barbanel D, Eldridge S, Griffiths C (2003). Can a self-management programme delivered by a community pharmacist improve asthma control? A randomised trial. Thorax.

[10] Hibbard JH, Mahoney ER, Stockard J, Tusler M (2005). Development and testing of a short form of the patient activation measure. Health Serv Res.

[11] Shah SL, Siegel CA (2015). Increasing patient activation could improve outcomes for patients with inflammatory bowel disease. Inflamm Bowel Dis.

[12] Cunha CM, da Cunha D, Manzato RO (2019). Validation of the Brazilian version of the Patient Activation Measure 13. J Nurs Meas.

[13] Oliveira GMM, Brant LCC, Polanczyk CA (2020). Cardiovascular Statistics – Brazil 2020. Arq Bras Cardiol.

[14] Rademakers J, Maindal HT, Steinsbekk A (2016). Patient activation in Europe: an international comparison of psychometric properties and patients’ scores on the short form Patient Activation Measure (PAM-13). BMC Health Serv Res.

[15] PAM Insignia Health 2024 PAM Insignia Health 2024.

[16] Wallston KA, Stein MJ, Smith CA (1994). Form C of the MHLC scales: a condition-specific measure of locus of control. J Pers Assess.

[17] Young L, Kupzyk K, Barnason S (2017). The impact of self-management knowledge and support on the relationships among self-efficacy, patient activation, and self-management in rural patients with heart failure. J Cardiovasc Nurs.

[18] Prochaska JO, DiClemente CC (1992). Stages of change in the modification of problem behaviors. Prog Behav Modif.

[19] Wilkinson TJ, Memory K, Lightfoot CJ, Palmer J, Smith AC (2021). Determinants of patient activation and its association with cardiovascular disease risk in chronic kidney disease: A cross-sectional study. Health Expect.

[20] Poon BY, Shortell SM, Rodriguez HP (2020). Patient activation as a pathway to shared decision-making for adults with diabetes or cardiovascular disease. J Gen Intern Med.

[21] Holter M, Avian A, Weger M (2024). Measuring patient activation: the utility of the Patient Activation Measure administered in an interview setting. Qual Life Res.

[22] Darabi F, Ziapour A, Mohamadkhah F (2025). Factors related to self-care behaviors’ in chronic heart failure patients: a cross-sectional study in Western Iran. Am J Health Promot.

[23] Dariya SS, Maheshwari A, Viswanathan V (2025). Assessment of the awareness of risk factors and current behavior among individuals with type 2 diabetes mellitus in India: a cross-sectional study. Cureus.

[24] Magnezi R, Glasser S, Shalev H, Sheiber A, Reuveni H (2014). Patient activation, depression and quality of life. Patient Educ Couns.

[25] Lunardi LE, K Le Leu R, Matricciani LA (2024). Patient activation in advanced chronic kidney disease: a cross-sectional study. J Nephrol.

[26] Goevaerts WF, Tenbült-van Limpt NCCW, Lu Y (2024). Evaluation of an application for the self-assessment of lifestyle behaviour in cardiac patients. Neth Heart J.

[27] Hernar I, Graue M, Igland J (2023). Patient activation in adults attending appointments in general practice: a cross-sectional study. BMC Prim Care.

[28] Zang Y, Wang L, Choi KC, Du H (2025). Impact of depression on activation and summer heat adaptation in older adults with cardiovascular concerns: empirical research quantitative. Nurs Open.

[29] Grafe W, Tinsel I, Borger M (2025). General practitioners’ attitudes and barriers to patient activation in cardiovascular disease prevention: insights from the DECADE study. BMC Prim Care.

[30] Nair D, Cavanaugh KL (2020). Measuring patient activation as part of kidney disease policy: are we there yet?. J Am Soc Nephrol.

[31] Tusa N, Kautiainen H, Elfving P, Sinikallio S, Mantyselka P (2020). Relationship between patient activation measurement and self-rated health in patients with chronic diseases. BMC Fam Pract.

[32] Janamian T, Greco M, Cosgriff D, Baker L, Dawda P (2022). Activating people to partner in health and self-care: use of the Patient Activation Measure. Med J Aust.

